# Potential and Metabolic Pathways of Eugenol in the Management of *Xanthomonas perforans*, a Pathogen of Bacterial Spot of Tomato

**DOI:** 10.3390/ijms232314648

**Published:** 2022-11-24

**Authors:** Mustafa Ojonuba Jibrin, Qingchun Liu, Timothy J. Garrett, Jeffrey B. Jones, Shouan Zhang

**Affiliations:** 1 Tropical Research and Education Center, University of Florida, IFAS, Homestead, FL 33031, USA; 2 Department of Pathology, Immunology, and Laboratory Medicine, University of Florida, Gainesville, FL 32610, USA; 3 Plant Pathology Department, University of Florida, Gainesville, FL 32611, USA

**Keywords:** tomato, *Xanthomonas perforans*, small molecules, eugenol, disease management, metabolites, molecular pathways

## Abstract

Bacterial spot of tomato continues to pose a significant problem to tomato production worldwide. In Florida, bacterial spot of tomato caused by *Xanthomonas perforans* is one of the most important diseases responsible for tomato yield loss. This disease is difficult to control, and new strategies are continually being investigated to combat the devastating effect of this disease. Recent efforts focusing on essential oils based on small molecules have spurred interests in the utilization of this class of chemicals for disease management. In this study, we evaluated the efficacy of eugenol for the management of bacterial spot of tomato caused by *X. perforans*. In the greenhouse experiments, eugenol applied as a foliar spray significantly (*p* < 0.5) reduced bacterial spot disease compared to the untreated control. In the field experiments, the area under the disease progress curve (AUDPC) was significantly (*p* < 0.5) lower in the plots treated with eugenol or eugenol combined with the surfactant Cohere than in the untreated control plots, and it was comparable to the copper-based treatments. To provide additional insights into the possible pathways of eugenol activities, we applied a liquid chromatography mass spectrometry (LC-MS)-based metabolomic study using a thermo Q-Exactive orbitrap mass spectrometer with Dionex ultra high-performance liquid chromatography (UHPLC) on *X. perforans* strain 91–118 treated with eugenol. Our results showed that eugenol affected metabolite production in multiple pathways critical to bacterial survival. For example, treatment of cells with eugenol resulted in the downregulation of the glutathione metabolism pathway and associated metabolites, except for 5-oxoproline, which accumulation is known to be toxic to living cells. While the peaks corresponding to the putatively identified sarmentosin showed the most significant impact and reduced in response to eugenol treatment, branched-chain amino acids, such as L-isoleucine, increased in production, suggesting that eugenol may not negatively affect the protein biosynthesis pathways. The results from our study demonstrated the efficacy of eugenol in the management of bacterial spot of tomato under greenhouse and field conditions and identified multiple pathways that are targeted.

## 1. Introduction

Small molecules are organic molecules with low molecular weight, often less than 900 Daltons, that are capable of permeating intracellular components where they interact and inhibit biological processes [1,2]. Many significant products of translation are short peptides that are critical to the functions of cells [3,4,5]. These short peptides are known as small molecules and constitute some of the most important molecules that function in biological systems and have been deployed in making novel drugs for managing important human diseases [2,6]. Recently, small molecules, some of which are essential oils, have been shown to hold promise in the management of plant diseases [7,8,9,10,11,12,13].

**Figure 1:**
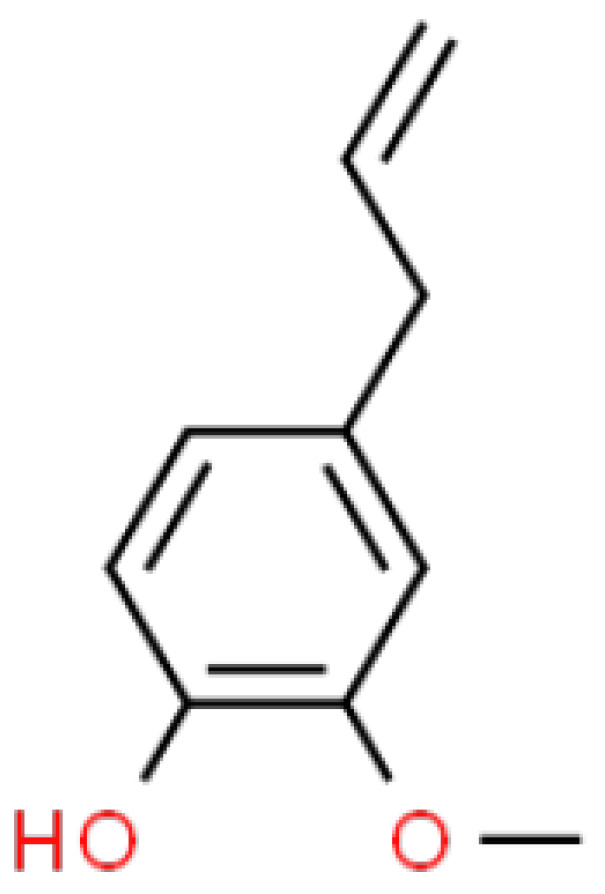
Chemical structure of the small molecule eugenol in essential oils, as obtained from ChemSpider (http://www.chemspider.com, accessed on 12 August 2022).

The phenylpropanoid pathway, also known as the cinnamic acid pathway, produces many small molecules and compounds which are collectively referred to as “cinnamic acids” [14]. The reduction of the carboxylic acid functional group of cinnamic acids results in the synthesis of monolignols, which subsequently yield another class of volatile organic compounds called phenylpropenes (C_6_–C_3_) [15]. Eugenol (4-allyl-2-methoxyphenol, C_10_H_12_O_2_; molecular weight 164.2 g/mol, Figure 1) is an example of phenylpropenes [15,16]. The phenylpropenes, eugenol and isoeugenol, are synthesized from coniferyl alcohol whose sidechains undergo acylation reactions before they are reduced in an NADPH-dependent manner [15,16]. Eugenol is a major component of essential oils originally extracted from plants in the family Myrtaceae, especially cloves including *Syzygium aromaticum* (L.) and *Eugenia caryophyllata* (L.) [17,18]. The eugenol biosynthesis pathway has been characterized in several plants, including *Arabidopsis thaliana*, *Clarkia brewri*, and *Ocimum basilicum* [19,20,21]. Eugenol is a volatile and colorless liquid and has chemical structure that is related to phenol [17,22]. It is classified as ‘Generally Recognized as Safe’ (GRAS) by the U.S. Food and Drug Administration (US FDA) as it is non-carcinogenic and non-mutagenic [23]. Eugenol has been demonstrated to have both antimicrobial activities and insecticidal properties [24,25]. For example, eugenol has been shown to permeate and destroy the integrity of cell membrane and inhibit biofilm formation of periodontal and foodborne pathogens [18,25,26]. It also has antioxidant, antimicrobial, anti-inflammatory, anti-cancer, and anti-diabetic activities [22,27]. 

Eugenol has also been shown to be effective against plant pathogens. For example, upon the exposure of *Botrytis cinerea* mycelia to eugenol, hydrogen peroxide and free Ca^2+^ were induced in the cytoplasm, suggesting its ability to bind to membrane and alter the permeability of membrane; this resulted in the destabilization and disruption of the plasma membrane [28]. Eugenol was also shown to induce peroxidase (POD), polyphenol oxidase (PPO), and phenylalanine ammonia lyase (PAL) in response to tomato yellow leaf curl virus (TYLCV) infection [29]. In addition, eugenol was shown to exhibit insecticidal properties against insects, such as *Sitophilus zeamais*, *Dinoderus bifloveatus*, and *Callosobruchus maculatus* [30,31,32]. Eugenol was previously shown to possess antibacterial activities against bacterial diseases caused by *Xanthomonas oryzae* pv. *oryzae*, *Xanthomonas citri* subsp. *citri*, and *Xanthomonas campestris* pv. *phaseoli* var. *fuscans* [33,34,35,36].

Bacterial spot of tomato causes economic losses worldwide and is caused by at least four species in the genus *Xanthomonas* [37,38,39]. These species include *X. euvesicatoria, X. perforans, X. gardneri,* and *X. vesicatoria* [39,40]. Bacterial spot affects above-ground plant parts, appearing initially as small, water-soaked lesions on leaves which subsequently coalesce to form large dark spots; these spots result in defoliation and sometimes even lesions on fruits [39]. This disease is historically difficult to control using conventional chemicals, and its durable resistance in tomatoes is yet to be identified and incorporated [39,40]. The occurrence of this disease soon after transplanting, accompanied with favorable climate conditions, could lead to total crop losses in pepper production [41]. In Florida State, USA, *X. perforans* is the dominant species and it is difficult to manage [42]. *X. perforans* is of global distribution, and rapid changes in its population structure as a result of recombination and race shifts are known to occur [39,43,44].

Recently, small molecules have been shown to improve the management of *X. perforans* in Florida [7,8,10,11]. Carvacrol, 3-indolylacetonitrile (IAN), and N-acetylcysteine (NAC) were shown to enhance the control of copper-resistant bacterial spot strains in south Florida [7,8,10,11]. Carvacrol improved disease management in greenhouse and field trials against *X. perforans* compared to the untreated control, with multiple pathways in the bacterium being altered [7,10,45]. IAN significantly reduced disease severity compared to the untreated control when applied three days prior to inoculation [8]. NAC similarly reduced bacterial spot severity when applied individually and in combination with copper bactericides in greenhouse and field trials [11]. A recent study further showed the pathways altered by NAC activities in *Xanthomonas citri* subsp. *citri*, suggesting the role of NAC in the downregulation of proteinogenic amino acids and its possible influence on protein synthesis [46]. In our study, the small molecule, eugenol, was evaluated for its potential in managing bacterial spot of tomato. Efforts were also made to provide insights into its pathway of activity in *X. perforans* using metabolomic approaches. The results from this study are important in laying a foundation for understanding the potentials of eugenol in the management of bacterial spot of tomato disease and its mechanisms of action in the bacterium.

## 2. Results

### 2.1. MIC, MBC, and Effects of Eugenol on Bacterial Spot in the Greenhouse

**Figure 2:**
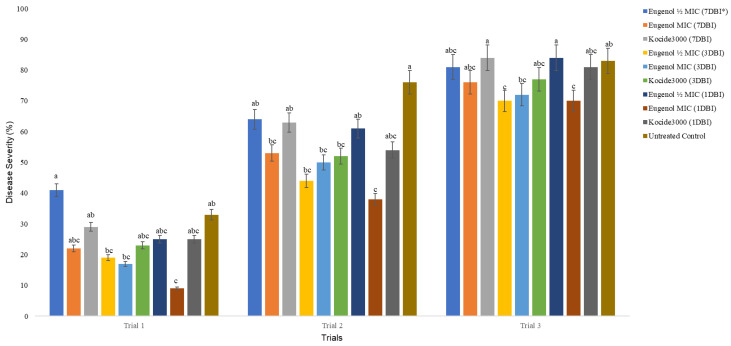
Effect of eugenol applied before inoculation on bacterial spot of tomato caused by *X. perforans*. The treatments are organized in each trial as the names appear from top to bottom on the side legend. * DBI = day before inoculation; MIC = minimum inhibitory concentration. The values in this figure are the ratings of disease severity. The error bars followed by the same letters in each trial are not significantly different (LSD_0.05_ for trials 1, 2, and 3 are 19.09, 22.74, and 11.94, respectively). The variation in disease severity observed between trials (trial 1 < trial2 < trial3) may be due to a sudden change in weather observed during the time conducting these trials, especially between trials 1 and 2.

In this study, the MIC and MBC for eugenol against *X. perforans* were 1024 mg/L across four replicates, indicating that approximately 1 g of eugenol in 1000 mL of ethanol:water (125:875) would inhibit the growth of *X. perforans*. The preliminary greenhouse experiments showed that the plants treated with eugenol one day before inoculation consistently had the lowest disease severity (*p* < 0.05), which was the only treatment that was consistently significantly different from the untreated plants (Figure 2).

### 2.2. Effects of Eugenol, Surfactants, and Combination of Eugenol and Surfactants Applied as a Foliar Spray against X. perforans in the Greenhouse

**Figure 3:**
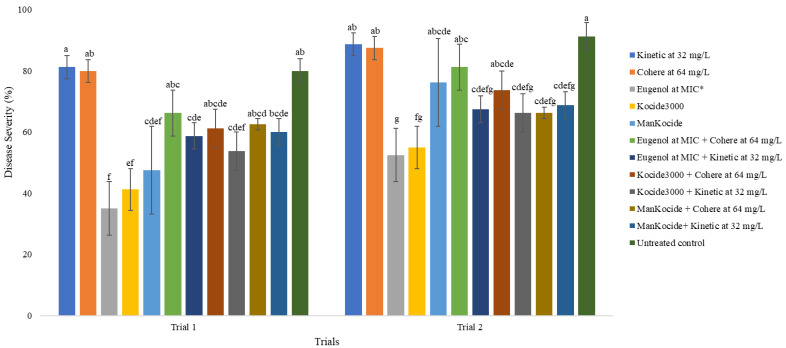
Effects of the combinations of eugenol with surfactants in the greenhouse on the control of bacterial spot of tomato. The treatments are organized in each trial as the names appear from top to bottom on the side legend. The minimum inhibitory concentration (MIC) of eugenol is 1024 mg/L. * The MIC of eugenol as used in this study is 1024 mg/L. The values in this figure are the ratings of disease severity. The error bars with the same letters in each column are not significantly different at *p* = 0.05.

The combinations of eugenol with each of the two surfactants, Kinetic and Cohere, were compared for their control of bacterial spot of tomato (Figure 3). In both trials, although the application of eugenol at the MIC significantly (*p* < 0.05) reduced disease severity compared to the untreated control, the effect was variable when eugenol was combined with the surfactants. In both trials, none of the combinations of Cohere with eugenol significantly reduced disease severity compared to the untreated control. However, the combination of eugenol at the MIC with Kinetic provided a significant reduction in disease severity compared to the untreated control in both trials.

### 2.3. Eugenol against Bacterial Spot of Tomato under Field Conditions

In the field trials, eugenol significantly (*p* < 0.05) reduced disease severity and the AUDPC compared to the untreated control (Table 1). The effects of this chemical were greater than or equivalent to Kocide3000 or ManKocide, the commercial standard controls in field trials. The plants treated with a combination of Cohere and eugenol or eugenol alone had significantly lower AUDPCs compared to the untreated control. Eugenol numerically increased the total fruit yield and extra-large fruit yield in both trials, albeit not significantly (data not shown).

### 2.4. Metabolic Effect of Eugenol against X. perforans as Revealed by LC–MS Metabolomics

**Figure 4:**
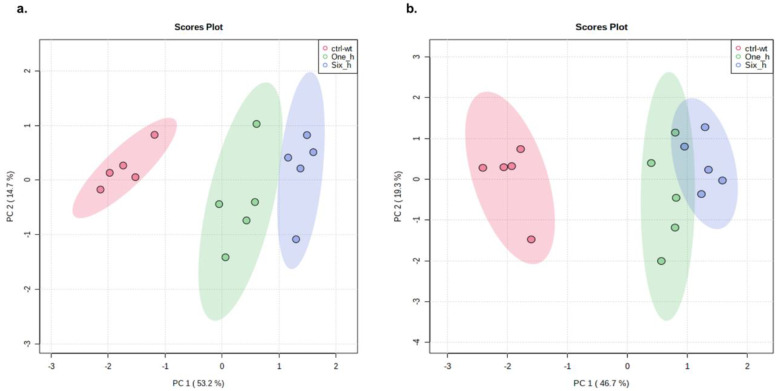
Graph of the principal component analyses for the annotated metabolites in (**a**) positive and (**b**) negative ion phases. Treated sa (Ctrl-wt = wild type *Xanthomonas perforans* Xp91–118; One_h = 1 h after treating Xp91–118 with eugenol; Six_h = 6 h after treating Xp91–118 treated with eugenol). The analyses were performed using MetaboAnalyst v. 5 [47] on 31 August 2022.

**Figure 5:**
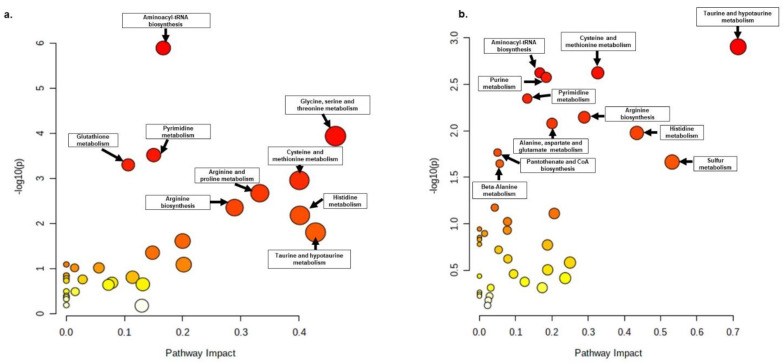
Potential pathways of significance of the annotated metabolites in both (**a**) positive and (**b**) negative ion modes after eugenol treatment of *X. perforans* 91–118. The metabolites are not targeted against the pathway of any organism. The pathways that show significance (*p* < 0.05) are shown. The gradual change in color of the circles from red to yellow and white shows the gradual decrease in the number of metabolites in each pathway (deep red circle represents many metabolites, while white represents no metabolites in the pathway). The analyses were performed using MetaboAnalyst v. 5 [47] on 31 August 2022. The metabolites in each pathway and their pattern of regulation are shown in Table 2.

**Figure 6:**
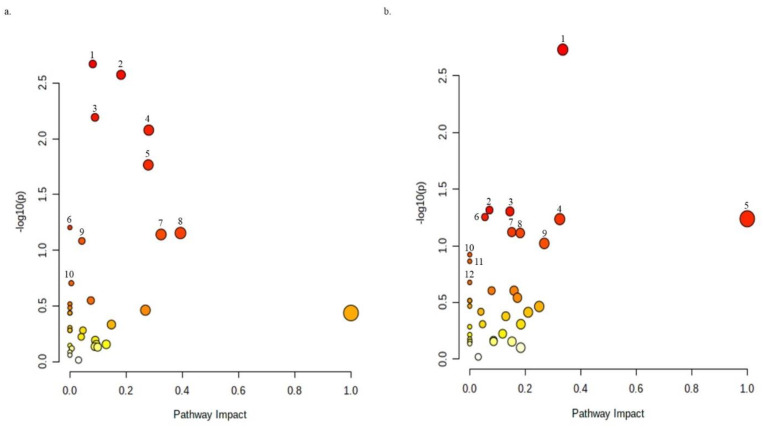
Simulated impacts of the annotated metabolites in both (**a**) positive and (**b**) negative ion modes after eugenol treatment of *X. perforans* 91–118. The simulation was carried out against the *Pseudomonas putida* KT2440 pathways. The top 10 and 12 pathways (*p* < 0.05) for both the positive and negative ion phases are shown. The gradual change in color of the circles from red to yellow and white shows the gradual decrease in the number of metabolites in each pathway (deep red circle represents many metabolites, while white represents no metabolites in the pathway). The pathways in the positive ion phase from 1–10 include glutathione metabolism; aminoacyl-tRNA biosynthesis; pyrimidine metabolism; arginine and proline metabolism; glycine, serine, and threonine metabolism; cyanoamino acid metabolism; cysteine and methionine metabolism; arginine biosynthesis; sulfur metabolism; and glyoxylate and dicarboxylate metabolism pathways. The pathways in the negative phase from 1–12 include sulfur metabolism; glyoxylate and dicarboxylate metabolism; purine metabolism; pyrimidine metabolism; taurine and hypotaurine metabolism; arginine biosynthesis; pantothenate and CoA biosynthesis; aminoacyl-tRNA biosynthesis; alanine, aspartate, and glutamate metabolism; monobactam biosynthesis; beta-alanine metabolism; and cyanoamino acid metabolism pathways. The analyses were performed using MetaboAnalyst v. 5 [47] (31 August 2022). The −log(*p*) and FDR for each shown pathway are available in Table 2.

Metabolite annotation was performed according to Level 2 of the Metabolomics Standards Initiative, as described previously [45]. The preliminary results of the one-way ANOVA after data normalization are shown in the Supplementary file (Figures S1a,b and S2a,b). The post hoc test implemented after the parametric one-way ANOVA showed that the highest significance (*p* < 0.05) was assigned to the peaks corresponding to sarmentosin degradation in both positive and negative ion phases (Figure S2a,b). The pattern hunter results further showed that sarmentosin degradation was positively and negatively correlated with diverse metabolites in the positive and negative ion phases (Figure S3). The principal component analyses showed that eugenol had a significant impact on the metabolome of *X. perforans* 91–118 (Figure 4). The Pearson correlation heatmap further suggested that Xp91–118 treated with eugenol was negatively correlated with untreated Xp91–118 (Figure S4a,b). The breadth of impacts for each annotated metabolite in both positive and negative ion phases are shown in Figure S5a,b. Both primary and secondary metabolites were significantly impacted by eugenol treatment. Most of the primary metabolites are amino acids, nucleosides, and their derivatives (Figure S5a,b). Apart from sarmentosin which is a cyanogenic glucoside, other secondary metabolites that were significantly affected by eugenol treatment are alkaloids, such as harman, norhaman, β-carboline, β-carboline-3-carboxylic acid, piperidine, (2S, 4S) monatin, and methoxybrassinin, among others (Figure S5a,b). Nontargeted and targeted pathway analyses were conducted for the annotated metabolites in both positive and negative ion phases independently, and the simulated impacts against the *Pseudomonas putida* KT2440 pathways identified 10 and 12 pathways that were strongly affected in the positive and negative ion phases, respectively (Figure 5 and Figure 6). The pathways and the putatively annotated metabolites involved in each pathway, as well as their regulation, are shown in Table 2.

## 3. Discussion

### 3.1. Eugenol Improves X. perforans Management in Greenhouse and Field Conditions

The small molecules 3-indolylacetonitrile, carvacrol, and N-Acetylcysteine have been shown to be effective in the management of bacterial spot of tomato in greenhouse and field conditions [7,8,10,11]. In this study, we conducted greenhouse and field trials to evaluate the potential of eugenol, a small molecule in essential oils, in the management of bacterial spot of tomato caused by *X. perforans*. Eugenol is an antioxidant, anti-inflammatory, and anti-bacterial chemical that has been used in medical sciences and plant protection [22,28,29,30,48]. The results from our study showed the prospects of using eugenol for the management of bacterial spot, an economically important disease of tomato in Florida and worldwide.

In this study, we showed that the MIC of eugenol in nutrient broth under the tested conditions was 1024 mg/L, indicating that the molecule has antibacterial activity against *X. perforans*. MIC can vary depending on the medium, the pathogen, the strain, or the solvents used [49,50]. Our results showed that approximately 1 g of eugenol in 1000 mL of ethanol: water (125:875) inhibited the growth of *X. perforans*. While our method sought to limit the influence of the solvent on eugenol activities [51], future work may be needed to improve eugenol activities and reduce the variation of eugenol mixture in this and other solvents. Interestingly, a previous study has shown the antibacterial activity of eugenol against *X. citri* subsp. *citri,* but not *X. fuscans* subsp. *aurantifolii* type B, suggesting that eugenol may not always show antibacterial activities against species within the genus *Xanthomonas* [35]. It is, therefore, important that the genetic background of the strains of pathogens be taken into consideration in the determination of MICs against other *Xanthomonas* species.

Eugenol is an important component of essential oils from many plants. Essential oils extracted from clove, cinnamon, thyme, and *Origanum* species have been shown to inhibit *X. vesicatoria* in vitro and in vivo, as well as when being used as seed treatment [52,53,54,55]. Eugenol has also been shown to reduce seed infection with *X. campestris* pv. *phaseoli* var. *fuscans* on bean seeds [56]. The results from our in vitro studies suggest that eugenol similarly has antibacterial effects against *X. perforans*. A previous study, however, showed that eugenol was effective when applied as a protective treatment 24 h before inoculation against tomato yellow leaf curl virus (TYLCV) [29]. Gene expression analyses in that study further confirmed that the activities of eugenol against TYLCV were primarily through induced resistance, as demonstrated by the increased transcription of the tomato PR-1 gene [29]. The in planta 7-day time-course greenhouse experiments consistently showed that eugenol applied 24 h before inoculation had the best effects on reducing the disease severity of bacterial spot in tomato plants (Figure 2). Whether this activity was due to antibacterial effects alone or a combination of antibacterial activities and induced resistance will need to be further investigated.

While the results from our greenhouse experiments confirmed the role of eugenol in managing bacterial spot of tomato, our study showed that great care must be taken when optimizing with surfactants. In this study, eugenol at the MIC level significantly reduced disease severity compared to the untreated control, but the effects of the combination with surfactants were not consistent, as was noted in previous studies [57]. We already showed in a previous study that the surfactants, Cohere and Kinetic, provided inconsistent results when applied individually against bacterial spot in the greenhouse, with Cohere providing encouraging disease control results in field evaluations [57]. Interestingly, in this study, the combination of Kinetic with eugenol at the MIC provided a significant reduction in bacterial spot of tomato compared to the untreated control, suggesting that Kinetic could be included in future field experiments to evaluate its efficacy under field conditions.

The results from the field trials in this study confirmed the potential effects of eugenol on reducing bacterial spot of tomato caused by *X. perforans* under field conditions. The efficacy of eugenol against *X. perforans* from the field trials is similar to that for other small molecules, such as 3-indolylacetonitrile, carvacrol, and N-acetylcysteine (NAC) [7,8,10,11]. Similarly, in previous study, the application of Cohere alone significantly reduced bacterial spot severity under field conditions and it was suggested for use in combination with other chemicals in disease management [57]. In this study, the combination of eugenol and Cohere also resulted in significantly lower disease severity and AUDPC than the untreated control and Kocide3000 in the field experiments.

### 3.2. Eugenol Regulates Metabolites in Pathways Critical for Bacterial Survival

Eugenol significantly affected multiple metabolites in *X. perforans* 91–118 metabolome, as shown by the principal component analyses. Interestingly, the most significantly impacted annotated metabolite, sarmentosin, is coregulated with multiple other metabolites, suggesting possible coregulation of multiple pathways in eugenol activities. Characterizing this putative sarmentosin pathway will be important in understanding eugenol activities in this system.

The pathway analyses suggested that eugenol targets several pathways in *X. perforans*. The pathways that were significantly regulated by the putatively annotated metabolites in both untargeted search and when targeted against the *Pseudomonas putida* KT2440 are widely present in Gram-negative bacteria, including *X. perforans* [45,46]. At least 10 pathways and 12 pathways were significantly affected by the putatively annotated metabolites in the positive and negative ion phases, respectively. Many of these pathways are integral to bacterial survival and the critical functioning of cells. For example, previous studies on untargeted metabolomics have linked glutathione to cell cycle progression in bacterial cells [58]. In our study, all but one metabolite in the glutathione pathway were expectedly downregulated at 6 h after eugenol treatment, when bacterial cells were dead. It was also not surprising that 5-oxoproline (pyroglutamic acid), which is known to cause severe anion gap metabolic acidosis at high levels of accumulation in living cells [59,60], was the only upregulated metabolite at 6 h in the glutathione pathway. Similarly, taurine and hypotaurine are known to be utilized by bacteria as the sole sources of carbon, nitrogen, and energy for growth [61]. Metabolites in the taurine and hypotaurine metabolism pathway were significantly regulated in this study, with both taurine and 3-sulfino-l-alanine being downregulated at 6 h; this was similar to the carvacrol activity in our previous study. In the purine pathway, the downregulation of most metabolites, apart from L-Glutamine, by eugenol treatment contrasted strikingly to the upregulation of the same metabolites that were shown in a previous study investigating carvacrol against the same Xanthomonas strain [45].

When compared to previous studies of small molecules and/or essential oils on *Xanthomonas* species, our results suggested that different essential oils might have similar or varying effects on bacterial systems. Both carvacrol and eugenol are small molecules in essential oils. In our previous studies, we showed the pathways of activities of metabolites regulated by carvacrol, which are largely similar to the pathways identified in this study [45]. However, some differences exist with respect to the upregulation or downregulation of individual metabolites in their respective pathways, for example, monophosphates in the purine pathways as stated previously. Interestingly, comparing the pathways affected by eugenol from this study to other studies utilizing the small molecule N-Acetylcysteine (NAC) against *Xanthomonas citri* subsp. *citri* showed similar results in the affected pathways [46]. This study and two previous studies utilizing carvacrol and NAC against *Xanthomonas* species showed that the pathways of glutathione metabolism; cyanoamino acid metabolism; sulfur metabolism; aminoacyl t-RNA metabolism; glyoxylate and decarboxylate metabolism; glycine, serine, and threonine metabolism; and alanine, aspartate, and glutamate metabolism were regulated by carvacrol, eugenol, and NAC [45,46]. It therefore appears that small molecules frequently target these pathways in *Xanthomonas* species. However, the pattern of metabolite regulation may be different. For example, while metabolites in the glutathione metabolism pathway were listed as upregulated in the effects of NAC against *X. citri* subsp. *citri,* most metabolites in this pathway were downregulated for the effects of carvacrol and eugenol on *X. perforans* [45,46]. Interestingly, isoleucine, a branched-chain amino acid (BCAA, which includes leucine and valine) which is an essential amino acid that regulates protein synthesis [62], showed upregulation in the treated strains in this study, suggesting that the pathway of protein synthesis may not be the primary target of eugenol activity. BCAAs were shown to be remarkably downregulated in previous studies utilizing resveratrol against *X. oryzae* pv. *oryzae* and NAC against *X. citri* subsp. *citri* [46,63].

## 4. Materials and Methods

### 4.1. Preparation of Stock Solutions of Eugenol

Stock solutions of eugenol (>99% GC; Tokyo Chemical Industry America, Portland, OR, USA) were prepared as previously described with slight modifications [10,57]. Eugenol is slightly soluble in water [64]. Therefore, 0.4 g of eugenol was weighed and transferred into 5 mL of ethanol to dissolve the chemical. Subsequently, sterile distilled water was added to each solution to a final of 40 mL. All stock solutions were stored at room temperature.

### 4.2. Determination of MIC and MBC of Small Molecules

All in vitro and greenhouse studies were conducted with strain QL1, an *X. perforans* strain isolated from symptomatic tomato leaves in Homestead, Florida [8]. The MIC (minimum inhibitory concentration) and MBC (minimum bactericidal concentration) of eugenol against *X. perforans* strain QL1 were determined as previously described [10,57]. Briefly, to determine the MIC for eugenol, two-fold serial dilutions were performed in a 96-well plate to obtain final concentrations of 16, 32, 64, 128, 256, 512, 1024, 2048, 4096, and 8192 mg/L of each chemical in the nutrient broth (NB). In addition, a suspension of strain QL1 at 1 × 10^8^ cfu/mL in the nutrient broth (NB) was prepared. For the well with the concentration of 8192 mg/L, 163.84 μL of the stock suspension of the chemical was mixed with 36.16 μL of the bacterium thoroughly. For the next nine wells, 100 μL from the preceding well with a higher concentration was mixed with 100 μL of the bacterial suspension at 10^8^ cfu/mL in successive wells. The controls were 100 μL of the bacterial suspension without any eugenol treatment and uninoculated NB (without any bacteria). Then, the 96-well plates were incubated at 28 °C for 24 h. The MIC was determined as the lowest concentration of eugenol which prevented the visible growth of the *X. perforans* strain QL1. Then, the bacterial suspensions at all concentrations with no visible bacterial growth from the 96-well plate were plated onto the NA plates. The minimum concentration with no bacterial growth on the NA plates was recorded as the MBC. Each experiment was performed in a separate 96-well plate, and the experiment was conducted 4 times.

### 4.3. Effects of Eugenol on X. perforans in the Greenhouse

The experiments were conducted in the greenhouse to determine the effect of eugenol on *X. perforans*. Tomato cultivar, Florida 47, grown on commercial soil potting mix (Miracle-Gro, Marysville, OH, USA) for up to three or four true-leaf stage was used for all greenhouse experiments. Based on the MIC of eugenol which was determined to be 1024 mg/L, eugenol was applied at that concentration and at ½ MIC (512 mg/L), and Kocide 3000 (at the label rate of 2.1 g/L) was applied as a standard commercial control. An untreated control without any chemical was included. There were five (5) plants per treatment. Each treatment was applied once. Since eugenol was previously reported to act by inducing resistance [29], treatments were applied at multiple days before inoculation (DBI) to further evaluate the best time at which the application of eugenol would give the optimal control. The treatments were, therefore, applied at seven (7) DBI, three (3) DBI, and one (1) DBI. The treatments were applied as foliar sprays on both sides of the leaves until run-off using a handheld sprayer. Twenty-four hours after the application of the last treatment (that is, 1 DBI), the treated and untreated plants were inoculated with *X. perforans* at 10^8^ cfu/mL by spraying on both surfaces of the matured leaves of each plant with a handheld sprayer until run-off. The inoculated plants were placed in a moist chamber and incubated overnight. Water was misted on the plants in the chamber to provide high humidity. The plants were removed from the moist chamber the next day after 15 h of incubation and maintained on the bench in the greenhouse where they were exposed to natural light for 12 h each of light and darkness. The plants were watered twice each day by both direct watering of the soil and sprinkling of water mist onto leaf surfaces in the morning and evening to maintain plant health and promote humidity. The ratings were performed starting from five (5) days after inoculation based on a visual estimation of the percentage of the leaf surface covered with bacterial spot lesions. The analysis was performed using ANOVA in R. The means were separated using LSD at *p* = 0.05. This experiment was conducted three times.

### 4.4. Effects of Eugenol and Combination with Surfactants against X. perforans

The greenhouse experiments were conducted to evaluate the effects of conventional organosilicon surfactants on the efficacy of eugenol against bacterial spot. We tested two surfactants, Kinetic and Cohere, whose MIC was previously determined to be 32 and 64 mg/L, respectively [57]. Cohere and Kinetic at the MIC level were applied as a foliar spray individually and in combination with eugenol at the MIC. Kocide 3000 at 2.1 g/L and ManKocide at 2.1 g/L, each with the surfactants, served as standard controls. An untreated control was also included. All treatments were applied once at 24 h before inoculation with *X. perforans*. There were fifteen plants per treatment. The treated plants were left for 24 h on the bench in the greenhouse at 27 °C. After 24 h, the treated and untreated plants were inoculated with the *X. perforans* strain QL1 at 10^8^ cfu/mL with a handheld sprayer. The inoculated plants were placed in a moist chamber and incubated overnight. Water was misted in the growth chamber to provide high humidity. The plants were removed from the chamber the next day after 15 h of incubation and placed on the bench in the greenhouse. The plants were watered twice each day by both direct watering of the soil and misting of water onto leaf surfaces in the morning and evening to maintain humidity. Disease ratings were made by assessing the percentage of leaf area covered with bacterial spot lesions. The statistical analysis was performed using ANOVA in R. The means were separated using LSD at *p* = 0.05. This experiment was conducted three times.

### 4.5. Field Evaluation of Eugenol for Management of Bacterial Spot on Tomato

Tomato (cv. Red Bounty) seedlings (3–4 weeks old) were transplanted into beds covered with plastic in October and December 2020 for trials 1 and 2, respectively. Each plot was 3.66 m in length on a single bed and consisted of 8 plants with 0.51 m between plants. The buffer zone between plots was 0.61 m. Treatments started at 2 weeks after transplanting and 8 applications were conducted in both trials following a weekly schedule. Silwet L-77 was added at a final concentration of 0.02% as a surfactant into both the treated plants and the untreated control. The plants were inoculated with a copper-tolerant *X. perforans* strain QL by spraying the bacterial suspension adjusted to 1 × 10^8^ CFU/mL on the leaf surface in the late afternoon the day before the third application of treatments. Disease severity was rated weekly when bacterial spot lesions were apparent on the untreated plants. Fruits were harvested three and two times in trials 1 and 2, respectively. The data on final disease severity, area under the disease progress curve (AUDPC), and fruit yield were analyzed using the SAS program (SAS9.4, Cary, NC, USA).

### 4.6. LC–MS Based Metabolomics Analyses of Effect of Eugenol on X. perforans

Metabolomic experiment and analyses were performed as previously described (please see [45] for extensive details). As in previous study, the *X. perforans* reference strain 91–118, isolated in 1991 in Florida [40], was used to provide a reference for future studies. Briefly, 24-h-old culture of *X. perforans* strain 91–118 was standardized at 10^8^ cfu/mL and treated with eugenol at the MIC level. The samples were taken at 1 and 6 h after treatment. The untreated samples were included as the control. All treatments and the control were replicated four times. A fifth sample that was pooled together from all four samples for each treatment was also included. The processed samples were analyzed at the Southeastern Center for Integrated Metabolomics (SECIM), University of Florida in Gainesville, FL, USA. Global metabolomic profiling was conducted using a Thermo Q-Exactive orbitrap mass spectrometer with Dionex UHPLC and autosampler (Thermo Fisher, San Jose, CA, USA). All samples were analyzed in positive and negative heated electrospray ionization with a mass resolution of 35,000 at *m*/*z* 200 as separate injections. All samples were injected as provided. Separation was achieved using an ACE 18-pfp 100 × 2.1 mm, 2 µm column (Mac-Mod Analytical, Chadds Ford, PA, USA) with mobile phase A of 0.1% formic acid in water and mobile phase B of acetonitrile. The flow rate was 350 µL/min with a column temperature of 25 °C, and 4 µL was injected for negative ions and 2 µL for positive ions. The data from the positive and negative ion modes were separately subjected to statistical analysis. All data were normalized to the sum of metabolites for each sample prior to analysis. MZmine v. 2 [65] (http://mzmine.github.io/, accessed on 10 January–30 August 2022) was used to identify features and deisotopes, and to align features and perform gap filling to fill in any features that might have been missed in the first alignment algorithm. All adducts and complexes were identified and removed from the data set. The data were searched against the SECIM internal retention time metabolite library of 1100 compounds for identification. Subsequent searches against HMDB, METLIN, and KEGG were carried out manually. MetaboAnalyst 5.0 (https://metaboanalyst.ca, accessed between 1 January 2022–30 August 2022) was used for both univariate, multivariate, and pathway analyses, as described previously [45,47].

## 5. Conclusions

In conclusion, the results from our present study confirmed the potential of eugenol for the management of bacterial spot of tomato caused by *X. perforans*. Eugenol demonstrated a significant reduction in bacterial spot of tomato in both greenhouse and field trials. The metabolomic studies showed that eugenol potentially affected multiple pathways in *X. perforans* that are essential for bacterial survival but may not be involved in targeting the protein synthesis pathways. While major pathways affected by the primary metabolites are shown in this studies, future studies focusing on the integration of the impact of secondary metabolites would provide additional insights into the global impacts of eugenol against *X. perforans*. Additionally, future studies focusing on the gene regulation of *X. perforans* in response to eugenol would provide insightful information on the specific targets of the chemical in bacteria and host plants, thereby improving the formulations of the chemical for potential commercial use. Development of effective formulations would be critical for their commercial use while avoiding non-targeted organisms.

## Figures and Tables

**Table 1:** Field evaluation of eugenol against bacterial spot of tomato.

Treatments	Trial 1		Trial 2	
	Severity	AUDPC	Severity	AUDPC
Untreated Control	19.0 a	212.6 a	49.4 a	560.3 a
Kocide3000	15.6 b	136.8 b	46.3 ab	475.0 b
ManKocide	13.1 bc	122.0 bc	44.2 bc	441.0 bc
Eugenol	12.1 c	108.0 c	42.3 bcd	424.1 cd
Eugenol + Cohere	11.7 c	104.6 c	38.5 d	380.2 d
LSD_0.05_	2.58	27.8	4.0	45.3

The means followed by different letters in each column are significantly different (*p* < 0.05) according to Fisher’s least significant difference. The values in this table are the ratings of disease severity. The means followed by the same letters in each column are not significantly different at *p* = 0.05.

**Table 2:** Significant pathways (*p* < 0.05) from the targeted pathway analyses using metabolites in Xp91–118 that are significantly regulated (*p* < 0.05) by eugenol treatment against the *Pseudomonas putida* KT2440 reference library. Fifteen annotated metabolites each in the positive and negative ion modes are without matches to the *P. putida* KT2440. The letters ‘U’ and ‘D’ following each metabolite indicate being upregulated and downregulated after 6 h, respectively, in Xp91–118 (Figure S5a,b).

S/N	Pathway Name	−log(*p*)	Metabolites Expressed in Regulated Pathways
Positive Ion phase			
1	Glutathione metabolism	2.6706	Glycine (D), L-Ornithine (D), Putrescine (D), 5-Oxoproline (U), L-Cysteine (D)
2	Aminoacyl-tRNA biosynthesis	2.5735	L-Glutamine (U), L-Cysteine, Glycine (D), L-Serine (U), L-methionine (U), L-Lysine (D), L-Isoleucine (U), L-Proline (D),
3	Pyrimidine metabolism	2.1917	Cytosine, L-Glutamine (U), CMP (D), Cytidine (U), Uridine (U), Uracil (D), Deoxycytidine (U)
4	Arginine and proline metabolism	2.0773	L-Ornithine (D), L-Proline (D), Creatine (D), Creatinine (U), Putrescine D), N-Acetylputrescine (U)
5	Glycine, serine, and threonine metabolism	1.7648	L-Serine (U), Glycine (D), L-Cystathionine (U), L-Cysteine, Creatine (D), Ectoine (U),
6	Cyanoamino acid metabolism	1.203	L-Asparagine (U), Glycine (D), L-Serine (U)
7	Cysteine and methionine metabolism	1.1538	L-Cystine (D), L-Serine (U), L-Cystathionine (U), L-Cysteine (D), L-methionine (U).
8	Arginine biosynthesis	1.1407	L-Glutamine (U), L-Citrulline, L-Ornithine
9	Sulfur metabolism	1.083	L-Serine (U), Taurine (D), L-Cysteine (D)
10	Glyoxylate and dicarboxylate metabolism	0.70377	Glycine (D), L-Serine (U), L-Glutamine (U).
Negative Ion Phase			
1	Sulfur metabolism	2.7316	L-Serine (U), Sulfate (D), Succinate (D), Sulfite (D), Taurine (D).
2	Glyoxylate and dicarboxylate metabolism	1.3151	(S)-Malate (D), L-Serine (U), L-Glutamine (U), Succinate (D)
3	Purine metabolism	1.3032	Hypoxanthine (D), Xanthine (D), L-Glutamine (U), D-Ribose-5-phosphate (D), Sulfate (D), GMP (D), AMP (D)
4	Pyrimidine metabolism	1.2534	L-Glutamine (U), CMP (D), Cytidine (U), Uridine (U), Uracil (D)
5	Taurine and hypotaurine metabolism	1.2383	Taurine (D), Sulfite (D)
6	Arginine biosynthesis	1.2352	L-Glutamine (U), L-Ornithine (U), L-Citrulline (U)
7	Pantothenate and CoA biosynthesis	1.1208	Uracil (D), Pantothenate (U), Pantetheine (D)
8	Aminoacyl-tRNA biosynthesis	1.1126	L-Histidine (U), L-Glutamine (U), L-Serine (U), L-Methionine (U), L-Proline (D)
9	Alanine, aspartate, and glutamate metabolism	1.0208	L-Asparagine (U), Succinate (D), L-Glutamine (U)
10	Monobactam biosynthesis	0.92173	L-Serine (U), Sulfate (D)
11	Beta-alanine metabolism	0.863	Uracil (D), Pantothenate (U)
12	Cyanoamino acid metabolism	0.67626	L-Asparagine (U), L-Serine (U)

## Data Availability

The input files and scripts for the metabolomics analyses in this paper are available at https://github.com/ojonuba/Eugenol-IJMS-Metabolomics-Analyses, accessed 21 November 2022.

## Supplementary Materials

The supporting information can be downloaded at https://www.mdpi.com/article/10.3390/ijms232314648/s1.
