# Supplementary material for: Potential and Metabolic Pathways of Eugenol in the Management of Xanthomonas perforans, a Pathogen of Bacterial Spot of Tomato
Source: Int J Mol Sci. 2022 Nov 24;23(23):14648. doi: 10.3390/ijms232314648 (PMC9739100; doi:10.3390/ijms232314648)
Supplement: Supplementary file 1 [file ijms-23-14648-s001.zip › ijms-1971961-supplementary.pdf]

Supplementary File 1

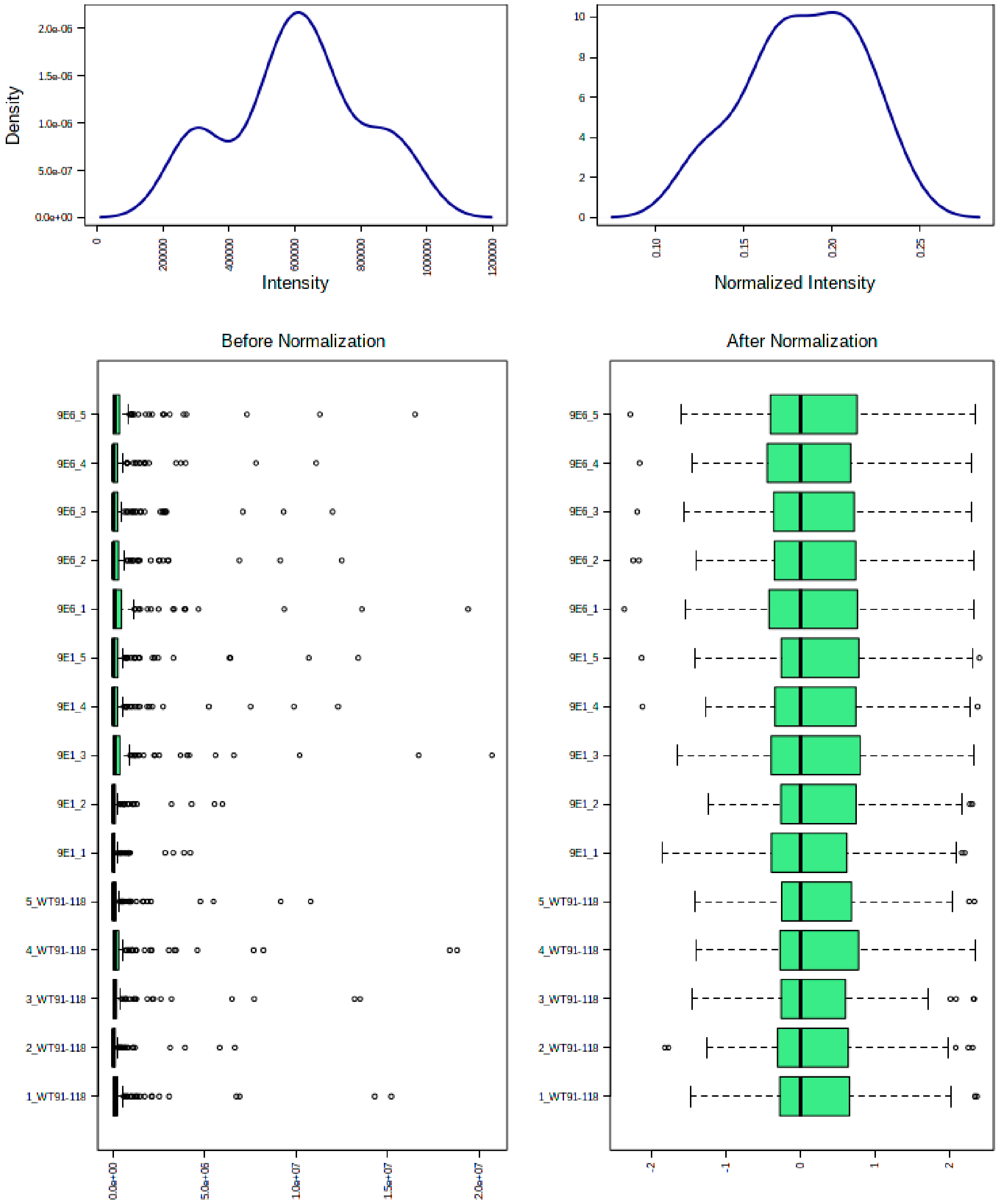

(a)

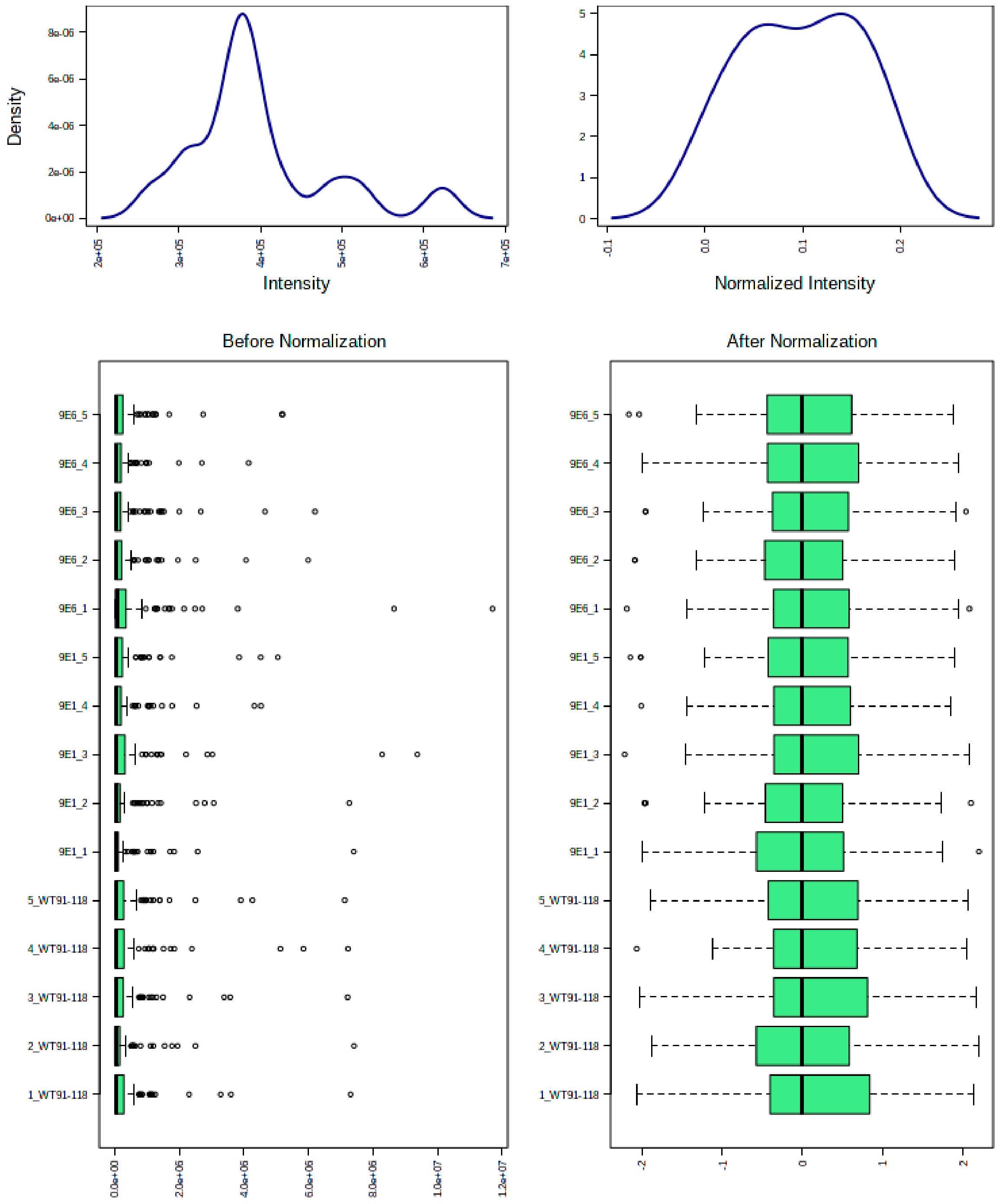

(b)

Figure S1. a. Normalized peak intensity for positive ion phase data (WT91-118=wild type Xp91-118; 9E1=1 hour after treating Xp91-118 with eugenol; 9E6= 6 h after treating Xp91-118 treated with eugenol). b. Normalized peak intensity for negative ion phase data. (WT91-118=wild type Xp91-118; 9E1=1 hour after treating Xp91-118 with eugenol; 9E6= 6 h after treating Xp91-118 treated with eugenol).

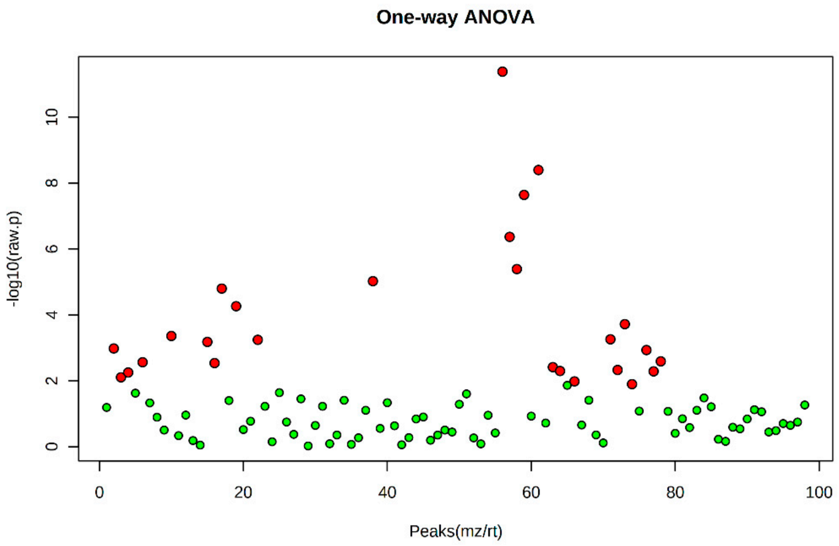

(**a**)

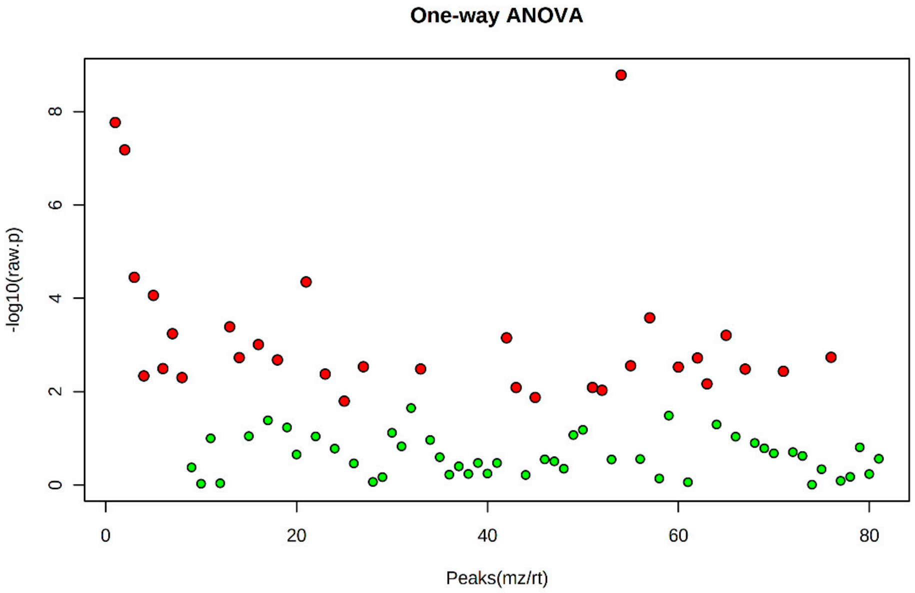

(**b**)

Figure S2. a. One-way ANOVA showing most significantly expressed peaks among the annotated metabolites in positive ion phase. The highest peak along the -log10(raw.p) axis in red correspond to the metabolite Sarmentonsin_276.1071-6.30. b. One-way ANOVA showing most significantly expressed peaks among the annotated metabolites in negative ion phase. The highest peak along the -log10(raw.p) axis in red correspond to the metabolite Sarmentonsin_276.1071-6.30.

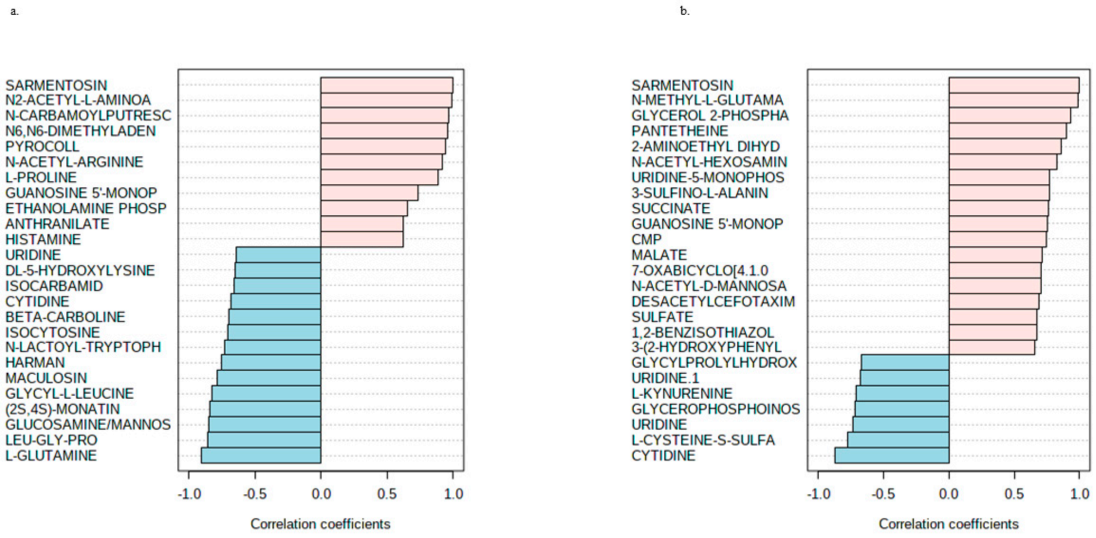

Figure S3. The top correlated annotated 25 metabolites with sarmentosin, the most significantly annotated metabolite (P<0.05) in the (a) positive and (b) negative ion phases.

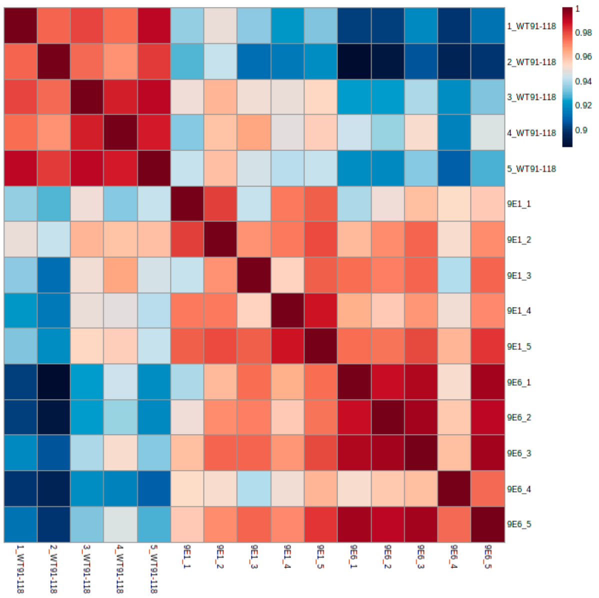

(**a**)

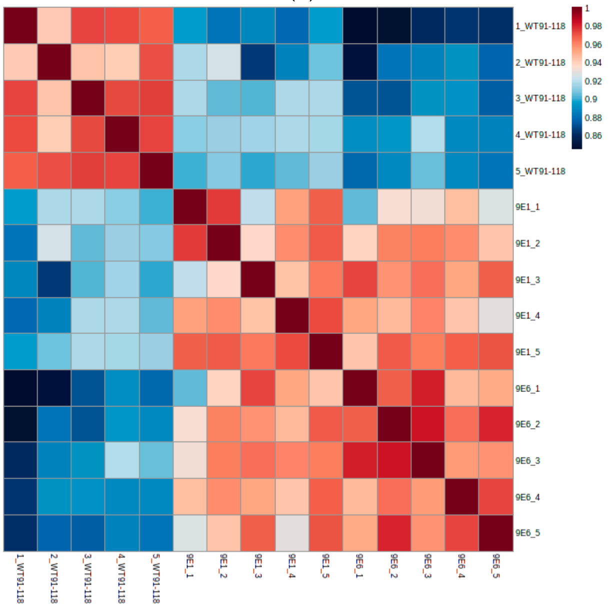

(**b**)

Figure S4. a. Correlation (Pearson r) heatmap of the annotated metabolites of the three treatments in positive ion phase. (WT91-118=wild type Xp91-118; 9E1=1 hour after treating Xp91-118 with eugenol; 9E6= 6 h after treating Xp91-118 treated with eugenol). b. Correlation (Pearson r) heatmap of the annotated metabolites of the three treatments in negative ion phase. (WT91-118=wild type Xp91-118; 9E1=1 hour after treating Xp91-118 with eugenol; 9E6= 6 h after treating Xp91-118 treated with eugenol).

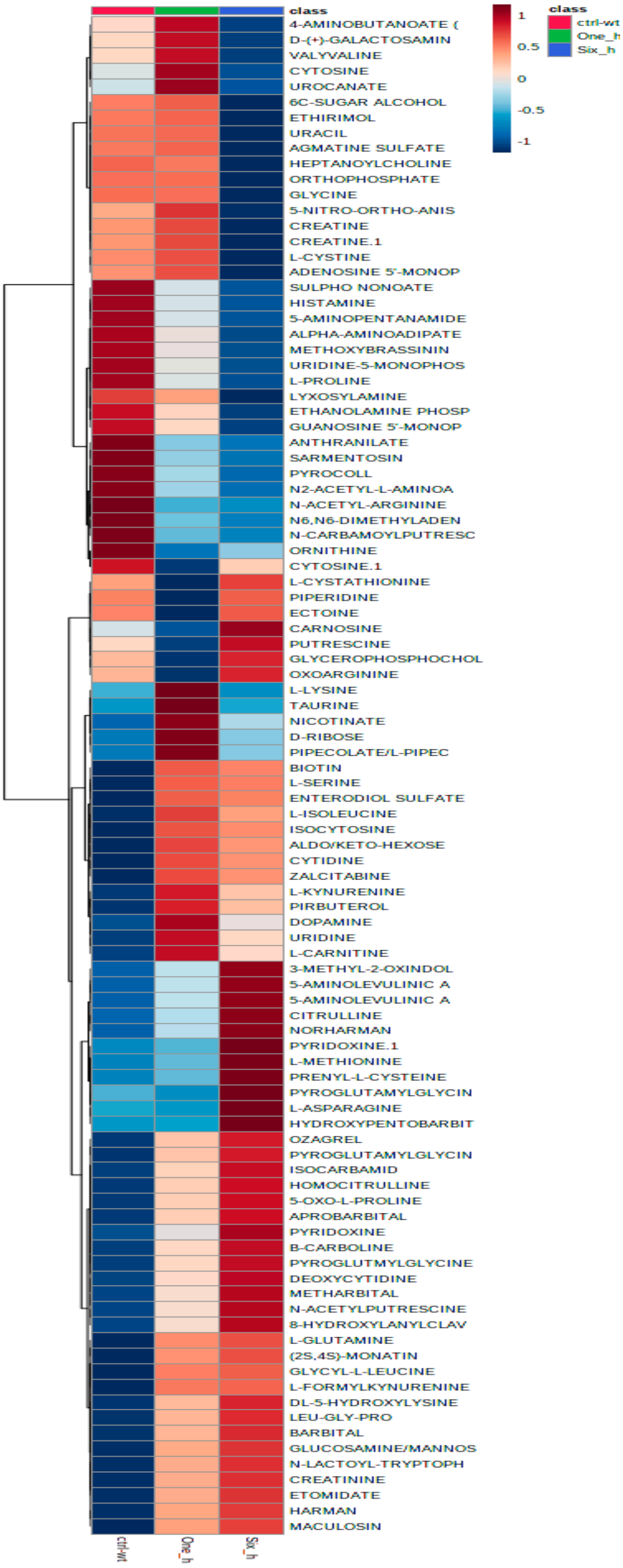
class
4-AMINOBUTANOATE (
D-(+)-GALACTOSAMIN
VALYVALINE
CYTOSINE
UROCANATE
6C-SUGAR ALCOHOL
ETHIRIMOL
URACIL
AGMATINE SULFATE
HEPTANOYLCHOLINE
ORTHOPHOSPHATE
GLYCINE
5-NITRO-ORTHO-ANIS
CREATINE
CREATINE.1
L-CYSTINE
ADENOSINE 5'-MONOP
SULPHO NONOATE
HISTAMINE
5-AMINOPENTANAMIDE
ALPHA-AMINOADIPATE
METHOXYBRASSININ
URIDINE-5-MONOPHOS
L-PROLINE
LYXOSYLAMINE
ETHANOLAMINE PHOSP
GUANOSINE 5'-MONOP
ANTHRANILATE
SARMENTOSIN
PYROCOLL
N2-ACETYL-L-AMINOA
N-ACETYL-ARGININE
N6,N6-DIMETHYLADEN
N-CARBAMOYLPUTRESC
ORNITHINE
CYTOSINE.1
L-CYSTATHIONINE
PIPERIDINE
ECTOINE
CARNOSINE
PUTRESCINE
GLYCEROPHOSPHOCHOL
OXOARGININE
L-LYSINE
TAURINE
NICOTINATE
D-RIBOSE
PIPECOLATE/L-PIPEC
BIOTIN
L-SERINE
ENTERODIOL SULFATE
L-ISOLEUCINE
ISOCYTOSINE
ALDO/KETO-HEXOSE
CYTIDINE
ZALCITABINE
L-KYNURENINE
PIRBUTEROL
DOPAMINE
URIDINE
L-CARNITINE
3-METHYL-2-OXINDOL
5-AMINOLEVULINIC A
5-AMINOLEVULINIC A
CITRULLINE
NORHARMAN
PYRIDOXINE.1
L-METHIONINE
PRENYL-L-CYSTEINE
PYROGLUTAMYLGLYCIN
L-ASPARAGINE
HYDROXYPENTOBARBIT
OZAGREL
PYROGLUTAMYLGLYCIN
ISOCARBAMID
HOMOCITRULLINE
5-OXO-L-PROLINE
APROBARBITAL
PYRIDOXINE
B-CARBOLINE
PYROGLUTMYLGLYCINE
DEOXYCYTIDINE
METHARBITAL
N-ACETYLPUTRESCINE
8-HYDROXYLANYLCLAV
L-GLUTAMINE
(2S,4S)-MONATIN
GLYCYL-L-LEUCINE
L-FORMYLKYNURENINE
DL-5-HYDROXYLYSINE
LEU-GLY-PRO
BARBITAL
GLUCOSAMINE/MANNOS
N-LACTOYL-TRYPTOPH
CREATININE
ETOMIDATE
HARMAN
MACULOSIN
ctrl-wt
One_h
Six_h
1
0.5
0
-0.5
-1
class
ctrl-wt
One_h
Six_h

(a)

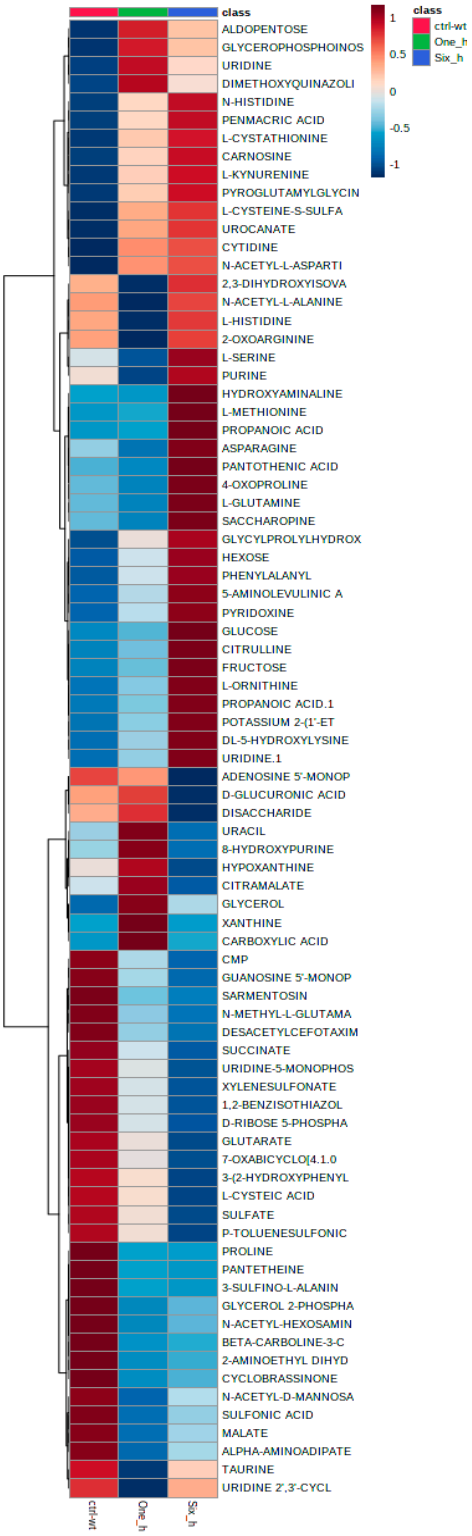

class
1
0.5
0
-0.5
-1
class
ctrl-wt
One_h
Six_h
ALDOPENTOSE
GLYCEROPHOSPHOINOS
URIDINE
DIMETHOXYQUINAZOLI
N-HISTIDINE
PENMACRIC ACID
L-CYSTATHIONINE
CARNOSINE
L-KYNURENINE
PYROGLUTAMYLGLYCIN
L-CYSTEINE-S-SULFA
UROCANATE
CYTIDINE
N-ACETYL-L-ASPARTI
2,3-DIHYDROXYISOVA
N-ACETYL-L-ALANINE
L-HISTIDINE
2-OXOARGININE
L-SERINE
PURINE
HYDROXYAMINALINE
L-METHIONINE
PROPANOIC ACID
ASPARAGINE
PANTOTHENIC ACID
4-OXOPROLINE
L-GLUTAMINE
SACCHAROPINE
GLYCYLPROLYLHYDROX
HEXOSE
PHENYLALANYL
5-AMINOLEVULINIC A
PYRIDOXINE
GLUCOSE
CITRULLINE
FRUCTOSE
L-ORNITHINE
PROPANOIC ACID.1
POTASSIUM 2-(1'-ET
DL-5-HYDROXYLYSINE
URIDINE.1
ADENOSINE 5'-MONOP
D-GLUCURONIC ACID
DISACCHARIDE
URACIL
8-HYDROXYPURINE
HYPOXANTHINE
CITRAMALATE
GLYCEROL
XANTHINE
CARBOXYLIC ACID
CMP
GUANOSINE 5'-MONOP
SARMENTOSIN
N-METHYL-L-GLUTAMA
DESACETYLCEFOTAXIM
SUCCINATE
URIDINE-5-MONOPHOS
XYLENESULFONATE
1,2-BENZISOTHIAZOL
D-RIBOSE 5-PHOSPHA
GLUTARATE
7-OXABICYCLO[4.1.0
3-(2-HYDROXYPHENYL
L-CYSTEIC ACID
SULFATE
P-TOLUENESULFONIC
PROLINE
PANTETHEINE
3-SULFINO-L-ALANIN
GLYCEROL 2-PHOSPHA
N-ACETYL-HEXOSAMIN
BETA-CARBOLINE-3-C
2-AMINOETHYL DIHYD
CYCLOBRASSINONE
N-ACETYL-D-MANNOSA
SULFONIC ACID
MALATE
ALPHA-AMINOADIPATE
TAURINE
URIDINE 2',3'-CYCL
ctrl-wt
One_h
Six_h

(b)

Figure S5. a. Heatmap showing variations in peak intensities of the annotated metabolites of the three treatments in positive ion phase. (Ctrl-wt=wild type Xp91-118; One_h=1 hour after treating Xp91-118 with eugenol; Six_h= 6 h after treating Xp91-118 treated with eugenol). b. Heatmap showing variations in peak intensities of the annotated metabolites of the three treatments in the negative ion phase. (Ctrl-wt=wild type Xp91-118; One_h=1 hour after treating Xp91-118 with eugenol; Six_h= 6 h after treating Xp91-118 treated with eugenol).
